# The Perceived Impact of The First UK COVID-19 Lockdown on Companion Animal Welfare and Behaviour: A Mixed-Method Study of Associations with Owner Mental Health

**DOI:** 10.3390/ijerph18116171

**Published:** 2021-06-07

**Authors:** Emily Shoesmith, Luciana Santos de Assis, Lion Shahab, Elena Ratschen, Paul Toner, Dimitra Kale, Catherine Reeve, Daniel S. Mills

**Affiliations:** 1 Department of Health Sciences, University of York, York YO10 5DD, UK; elena.ratschen@york.ac.uk; 2 School of Life Sciences, University of Lincoln, Lincoln LN6 7DL, UK; lassis@lincoln.ac.uk (L.S.d.A.); dmills@lincoln.ac.uk (D.S.M.); 3 Department of Behavioural Science and Health, University College London, London WC1E 7HB, UK; lion.shahab@ucl.ac.uk (L.S.); dimitra.kale.09@ucl.ac.uk (D.K.); 4 School of Psychology, Queen’s University Belfast, Belfast BT9 5BN, UK; p.toner@qub.ac.uk (P.T.); c.reeve@qub.ac.uk (C.R.)

**Keywords:** human-animal interaction, human-animal relationships, companion animals, animal welfare, animal behaviour, COVID-19, mental health, loneliness

## Abstract

Background: Companion animals may be a positive presence for their owners during the COVID-19 pandemic. However, the welfare of a companion animal is strongly influenced by the behaviour of their owners, as well as their physical and social environment. We aimed to investigate the reported changes in companion animal welfare and behaviour and to examine the association between these changes and companion animal owners’ mental health. Methods: A cross-sectional online survey of UK residents over 18 years of age was conducted between April and June 2020 (n = 5926). The questionnaire included validated, bespoke items measuring outcomes related to mental health, human-animal bonds and reported changes in animal welfare and behaviour. The final item of the survey invited open-ended free-text responses, allowing participants to describe experiences associated with human-animal relationships during the first UK lockdown phase. Results: Animal owners made up 89.8% of the sample (n = 5323), of whom 67.3% reported changes in their animal’s welfare and behaviour during the first lockdown phase (n = 3583). These reported changes were reduced to a positive (0–7) and negative (0–5) welfare scale, following principal component analysis (PCA) of 17 items. Participants reported more positive changes for cats, whereas more negative changes were reported for dogs. Thematic analysis identified three main themes relating to the positive and negative impact on companion animals of the COVID-19 pandemic. Generalised linear models indicated that companion animal owners with poorer mental health scores pre-lockdown reported fewer negative changes in animal welfare and behaviour. However, companion animal owners with poorer mental health scores since lockdown reported more changes, both positive and negative, in animal welfare and behaviour. Conclusion: Our findings extend previous insights into perceived welfare and behaviour changes on a very limited range of species to a wider range of companion animals. Owner mental health status has a clear, albeit small, effect on companion animal welfare and behaviour.

## 1. Introduction

Research on human-animal relationships suggests that companion animals can be a source of social support for their owners and help them cope with difficult situations [1,2,3]. In line with the British Small Animal Veterinary Association (BSAVA), companion animals in the UK are defined as ‘any domestic-bred or wild-caught animals, permanently living in a community and kept by people for company, enjoyment, work or psychological support—including, but not limited to dogs, cats, horses, rabbits, ferrets, guinea pigs, reptiles, birds and ornamental fish’. The first COVID-19 lockdown phase in the UK offered an opportunity to explore the role of companion animals as a source of emotional support. However, as research accumulates on the impact of companion animals on their owners during the pandemic [4,5,6,7], there has been a dearth of literature that focuses specifically on the welfare and behaviour of companion animals [8], and this may be quite species specific in its focus [9]. Most dog owners reported their dog’s routine had changed compared to pre-lockdown, and a reduction in the frequency of dog walking [9]. However, different species have different needs, so there is a need to appreciate the differential impact on diverse species.

Companion animals can experience negative consequences directly from confinement [10,11], but their quality of life is also directly influenced by the behaviour of their owners, and indirectly by their control of the physical and social environment of the species; factors which would be substantially affected during a lockdown period [12]. The COVID-19 pandemic may exacerbate these factors for multiple reasons [13]; for example, when owners are either furloughed or working from home for a prolonged period of time. This might lead to animals becoming frustrated or anxious at not being able to establish ‘quiet’ areas in the home where they would otherwise seek refuge [14]. Additionally, exercise routines for animals, primarily horses and dogs, may also be disrupted [9,14]. Furthermore, existing behavioural problems may be exacerbated or become more noticeable. Previous research has indicated that the behaviour modification plans of dog owners with pre-existing behavioural problems (e.g., anxiety, fearfulness or lack of socialisation) was disrupted by the pandemic [15]. There has also been restricted access to animal-related services (e.g., veterinary assistance, behavioural consultations, training classes, restricted access outdoors), which may impact on the development of behavioural problems [9,15].

Previous research has indicated that companion animal owners have expressed concerns about changes in their animal’s welfare and behaviour during the confinement period [3,8,13]. It is possible that these perceived changes reflect the owner’s underlying state of worry as a result of the COVID-19 pandemic. For example, if an individual is experiencing greater anxiety due to the current circumstances, they may project more anxiety onto their evaluation of their animal’s behaviour. Evidence suggests that highly anxious individuals are more likely to report greater concern about their animals [16]. This should be further investigated, given that the COVID-19 outbreak has resulted in substantial uncertainty, and fear of the unknown is a fundamental component of anxiety-related disorders [17].

Therefore, we investigated the following research questions:

1. What changes in companion animal welfare and behaviour do companion animal owners’ report during the first UK COVID-19 lockdown? (RQ1)
2. What are companion animal owners’ perceptions regarding the impact of the first UK COVID-19 lockdown on companion animal welfare and behaviour? (RQ2)
3. Do reported changes relating to animal welfare and behaviour differ by animal species? (RQ3)
4. What is the association between companion animal owners’ mental health scores pre-lockdown and since lockdown, and the reported changes relating to their animal’s welfare and behaviours? (RQ4)

## 2. Materials and Methods

### 2.1. Study Design

This study was a cross-sectional, retrospective survey, including free-text responses to an open-ended item.

### 2.2. Setting and Participants

The survey was conducted in the UK general population. All UK residents over 18 years of age were eligible to take part, irrespective of companion animal ownership. However, for the purpose of the current study, we focus on companion animal owners only. Companion animal owners owned a range of species, including dogs, cats, small mammals, birds, fish, reptiles or amphibians, horses or ponies, or farm animals.

### 2.3. Recruitment and Procedures

The survey was released in Qualtrics survey software and promoted using academic and third sector networks (including animal charities with an interest in human-animal interaction research), social media (Facebook, Twitter) and other media outlets (e.g., Reddit). The study commenced on 16 April 2020, four weeks after the first strict social distancing and social isolation measures came into force in the UK, and ended on 14 June, when the first lockdown measures were officially eased. Prospective participants followed a link to the survey where they were presented with a Participant Information Sheet and consent form. Consent to participate in the anonymous survey was indicated by ticking an online check box. A screening question requiring participants to name their country of residence denied access to non-UK residents. All data were stored on the secure Qualtrics server at the University of York.

Ethical approval for the survey was granted initially by the Health Sciences Research Ethics Committee at the University of York, UK on 16 April 2020.

### 2.4. Measures

As described in detail elsewhere [6], a bespoke questionnaire was developed by a multi-disciplinary team of academics with input from third sector animal welfare and training organisations. The questionnaire included validated items and new items based on expert consensus relating to emerging COVID-19-related aspects with reference to both companion and non-companion animals (e.g., wildlife). We provide a brief overview of the measures included in the current paper:

Demographics: Demographic information was gathered about participants’ age (in bands, including 70 and above), and gender (male/female/non-binary), used as covariates for RQ4.

Companion animal ownership: Participants were asked: ‘Do you have any animals that live with you or near you, and that you or anyone in your household are the main caretaker of? Please do not include animals kept as livestock (e.g., farm sheep, cattle).’ If answering ‘yes’, they were asked to indicate how many and which species (dog, cat, small mammal, bird, fish, reptile or amphibian, horse or pony, farm animal, other).

Respondents were asked to identify the animal they felt closest to, provide details of the species, and answer the remaining questions in relation to this companion animal. Respondents were asked to indicate whether this animal was an assistance dog or another form of working dog, a therapy animal or an emotional support animal. A response option ‘none of the above’ was provided. The variable relating to this question was conceptualised as ‘animal role’ for the purpose of the analyses and treated as a covariate for RQ4.

Perceived changes in companion animal’s welfare and behaviour: Companion animal owners were asked to indicate whether they agreed with the 17 statements (yes/no) relating to changes in their animals’ behaviour and welfare during the first COVID-19 lockdown phase in the UK. For example, ‘my animal seems more relaxed’, ‘my animal seems more anxious/easily scared’, and ‘my animal’s physical condition (e.g., coats/feathers) seems to have improved’. There was also an additional option to indicate that there had been no changes in their companion animal’s behaviour. The measure with the full range of response options is presented in Supplementary Table S1. The 17 statements were reduced to create subscales used as outcome variables for RQ4.

Human-animal bond and interactions: Companion animal owners were asked to indicate agreement to statements on the validated 11-item Comfort from Companion Animals Scale (CCA) [18], using a four-point Likert scale (1 = strongly disagree; 4 = strongly agree). Scores for each item on the CCA were combined into one total score (11–44) and included as a covariate for RQ4. As described in detail elsewhere [6], we refer to this measure as an instrument that measures the comfort or ‘closeness/intimacy’ dimension of the human-animal bond.

A single item asking participants to indicate whether they perceived their companion animal as a ‘member of the family’ was also asked, using the same four-point Likert scale (1 = strongly disagree’ 4 = strongly agree), and included as an ordinal covariate for RQ4.

Mental health: The mental health subscale of the SF-36 (MHI-5) [19] was included, with higher scores representing better mental health. The MHI-5 scale was used to collect current and retrospective data, asking participants to indicate their perceptions for the time ‘before lockdown’ and the present time at questionnaire completion (during the lockdown phase). Mental health scores pre-lockdown and since lockdown were used as predictors for RQ4.

Free-text responses: As described elsewhere [3], the survey included an option for participants to leave an open-ended, free-text comment to describe their experiences and perceptions of their human-animal relationships and interactions during the COVID-19 lockdown phase in the UK.

### 2.5. Data Analysis

In order to address RQ1, descriptive summary statistics are presented for data relating to the reported changes in companion animal welfare and behaviour during the first COVID-19 lockdown phase in the UK.

In order to address RQ2, responses to the free-text question that related specifically to animal behaviour and welfare were exported to QSR NVivo 12 software. The free-text comments were analysed using thematic analysis [20], employing an inductive approach, in which coding and theme development were driven by the content of the responses. One author (E.S.) familiarised herself with the data by reading all responses related to animal behaviour and welfare, and notes were made of any potential codes by identifying recurring words or units of meaning. The same author generated initial codes from the data and organised them into meaningful groups. Codes were then organised into potential themes and all relevant coded responses were collated within the identified themes. Two authors (E.S. and D.K.) independently reviewed the construction of themes and relevant quotations to agree to the assignment of themes.

As the item relating to animal welfare and behaviour included seventeen responses, a principal component analysis (PCA) with Varimax rotation was performed to reduce the number of responses into workable constructs to address RQ3 and RQ4. PCA is a data reduction method to simplify data into unique components [21]. The Kaiser-Meyer-Olkin (KMO) measure of sampling adequacy was performed to assess the merit of performing a factor analysis. The KMO is measured from 0 to 1 with acceptance of a value > 0.5 [22]. Parallel analysis was used to determine the number of principal components (PCs) extracted, with individual component loadings of > 0.4 used for interpretive purposes. Five responses did not load > 0.4, and were excluded from further consideration. Cronbach’s alpha coefficient was used to calculate the internal consistency of each PC. This process resulted in two welfare subscales; a positive (PC1; 0–7 items) and negative scale (PC2; 0–5 items).

Each item making up the welfare subscales were scored as either a “1” or “2” (absent or present, respectively), and multiplied by its loading within a given welfare component. All relevant items (loading > 0.4) were then summed and divided by the total possible score for the specific PC to generate a score within a standardised range between 1 to 2. Therefore, all species had PC scores relating to their welfare, which could be used in further analysis.

In order to address RQ3, animal species were compared and a non-parametric one-way MANOVA was conducted to assess whether the PC scores differed significantly between species, followed by post-hoc Dunn’s test with Bonferroni correction for multiple comparisons.

To address RQ4, generalised linear models were constructed. Gamma distribution was used due to the response variables (PCs) being positively, continuous and negatively skewed. These models assessed the association between each predictor (mental health score pre-lockdown and mental health score since lockdown), and the reported animal welfare and behaviour changes (PC1 and PC2), adjusting for relevant covariates (age, gender, animal role, human-animal bond measured by the continuous total score of the CCA, and the single item asking participants to identify whether they perceived their animal as a ‘member of the family’). The human-animal bond was included as a covariate, as evidence has reported that a stronger bond is associated with the types of concerns expressed [23]. The Cox and Snell pseudo R^2^ was calculated for each model.

Statistical analysis was implemented with R version 4.0.2 (R Core Team, Vienna, Austria) [24]. Standard alpha-levels were applied in two-tailored tests of significance (*p* < 0.05 considered significant). All analyses were pre-specified and uploaded on the Open Science Framework: https://osf.io/rnv6p/ (accessed on 2 February 2021).

## 3. Results

A total of 5926 participants consented and were eligible to take part in the study. Of 5926 participants, 5323 (89.8%) had at least one companion animal (see Table 1 for companion animal owner characteristics), and participants could report owning more than one species. The complete participant characteristics for the full survey sample are provided elsewhere [6].

### 3.1. What Changes in Companion Animal Welfare and Behaviour do Companion Animal Owners’ Report during the First UK COVID-19 Lockdown? (RQ1)

Nearly a third of companion animal owners (32.7%) reported that there had been no changes in their animal’s welfare and behaviour during the first COVID-19 lockdown phase.

Of companion animal owners who did report changes in companion animal welfare and behaviour since the lockdown phase started, a third (33.1%) reported their animal had been following them around more (primarily dog and cat owners), and just over a quarter (27.5%) said their companion animal had been more affectionate. However, only 11.0% of participants said their animal seemed more unsettled, and 5.9% identified their animal appeared more anxious or easily scared. Table 2 presents the complete reported changes in the welfare and behaviour of companion animals perceived as closest during the first COVID-19 lockdown phase, grouped by dogs, cats, horses and companion farm animals, and others (small mammals, birds, fish, reptiles, amphibians, and others).

### 3.2. What Are Companion Animal Owners’ Perceptions Regarding the Impact of the First UK COVID-19 Lockdown on Companion Animal Welfare and Behaviour? (RQ2)

Of 934 participants who provided a response to the optional free-text item, 828 (88.7%) were companion animal owners. The full participant characteristics for this sub-sample are presented elsewhere [3]. The thematic analysis of free-text responses, many of which included substantial detail and were characterised by narrative depth, resulted in the identification of three main themes with associated sub-themes related to various aspects of animal welfare and behaviour during the first COVID-19 lockdown phase (see Table 3). To illustrate themes and sub-themes, free-text responses are presented as *verbatim* quotes below, with the gender and age range of participants provided in brackets.

#### 3.2.1. Positive Impact on Companion Animals during COVID-19

##### Improvement in Animal’s Behaviour and Temperament

Many participants commented that there had been a positive change in their animal’s temperament. For example, some animals appeared to have become more settled than they were prior to the COVID-19 lockdown phase:

*“I have two rescue cats—one was very skittish, but she is much calmer now I am home every day.”*

(female, 65–70)

*“My dogs seem to feel happier that deliveries do not need to be signed for and are left on the drive due to COVID-19. I think they must have felt threatened by strangers coming to the door.”*

(female, 45–54)

*“The lack of manic lifestyle seems to make them *[dog and cat]* more settled.”*

(female, 45–54)

One participant perceived a positive change in her dog but acknowledged the reason for this may be because she had more time during the lockdown phase to train her.

*“In many ways, our dog has benefitted massively from having us around more. Her behaviour and temperament is improving every day because we are around to train her.”*

(female, 45–54)

##### Improvement in Animal’s Physical Condition

A small number of participants commented on the positive changes in their animal’s physical condition. All of those who reported improved changes in the free-text response were either dog or cat owners.

*“He *[my dog]* is playing more, and his coat seems shinier than ever before.”*

(female, 45–54)

*“Our cats have never been better groomed.”*

(female, 55–64)

#### 3.2.2. Negative Impact on Companion Animals during COVID-19

##### Concerns over Changes in Animal’s Temperament

Some participants reported that the first COVID-19 lockdown phase had resulted in a negative change of temperament in their companion animal. Of these, it was frequently expressed that their companion animal had become *‘needy’*, and some participants noted their animal was experiencing separation-related problems when they left the house, even for short periods of time.

*“My dog has become a lot more needy and howls if I leave the house without him, even if it’s just to do some gardening and he can see me. Going back to work will be very hard on him.”*

(female, 45–54)

*“My dog has become clingier to my husband and wants to sit with him on his return from work. She licks him more than before and wants to be stroked by him.”*

(female, 45–54)

One participant reported that although there were positive changes in her cat’s temperament at the beginning of the COVID-19 lockdown phase, this had progressively changed over time. The participant noted that the negative change in one cat’s temperament had subsequently had an impact on both the human-animal relationship, and the relationship between the two cats.

*“I have two cats, and both have acted very differently since I have been home. At first they were very excited, following me around and very affectionate and playful, but in recent weeks, they seem to have grown bored of my presence and interact with me much less unless they want something. One has become very temperamental and is often in a bad mood with me, and the other cat”.*

(female, 25–34)

Additionally, one participant noted that the perceived change in her animal’s behaviour had resulted in a negative effect on her family:

*“My dogs behaviour has changed dramatically and in turn, has had a negative effect on the family. I have even considered employing a dog trainer.”*

(female, 45–54)

##### Concerns over Changes in Animal’s Physical Condition

A small number of participants expressed concerns about changes in their animal’s physical condition. These participants were primarily dog owners, and highlighted concerns in relation to weight gain due to dog walking restrictions.

*“As we are only allowed out for a certain amount of time for exercise, this has considerably shortened the amount of time spent dog walking. I am anxious about this as my dogs have been putting on weight.”*

(male, 45–54)

*“Lack of exercise is the biggest problem. I used to walk three times a day, and now restricted to one period of exercise and I am having to alternate days as I can’t walk all six at once. I have to reduce the length of the walk to accommodate an older dog on her day. The weight gain caused by this concerns me.”*

(female, 65–70)

#### 3.2.3. Broader Impact of COVID-19 on Animal Welfare

##### Negative Impact of Dog Walking Restrictions

Dog owners frequently mentioned that the restrictions to time spent outdoors and the social distancing measures in place had a negative impact on their dogs. Owners often reported that due to these restrictions, their dogs were having less exercise (as outlined in the theme above) and missed the interaction with other dogs and dog walkers.

*“My dog misses playing with other dogs that we meet on our walks.”*

(female, 55–64)

*“My dogs are very sociable with people and other dogs. They seem to find it hard to understand why this has stopped and other people/dogs cannot interact with them. While on a walk, they will stop and look at others we see and are much more distracted by them walking by than before.”*

(female, 55–64)

*“My dog misses the socialising; he doesn’t understand what has happened. He is a very friendly Labrador and doesn’t understand why people won’t make a fuss of him anymore, people cross the road to avoid him.”*

(female, 55–64)

Additionally, a number of participants commented that they had been feeling anxious or uncomfortable to walk their dogs in local areas when they were busy, significantly restricting the amount of exercise time for their dog.

*“More people are out and about exercising, so it’s been a lot harder to find spaces where I feel safe to walk the dogs. This has meant less exercise for the dogs and a little weight gain for them.”*

(female, 35–44)

##### Adoption and Fostering Considerations and Concerns

Several participants expressed that they were considering adopting or fostering a companion animal prior to the pandemic. However, many highlighted this was no longer possible due to the restrictions in place and many of the rescue centres had temporarily closed. Some participants reported that they intended to adopt or foster a companion animal as soon as the restrictions had been eased.

*“I was considering getting another rescue cat or dog but have decided not to for now due to the uncertainties resulting from the COVID-19 crisis.”*

(male, 70+)

*“We will rescue when our local cat rescue centre opens, as currently closed through Covid crises.”*

(male, 45–54)

*“I’ve thought about buying a dog for a few years, but due to COVID-19, I am considering this possibility more seriously.”*

(male, 18–24)

*“We had decided we wanted to adopt another rescue kitten before COVID-19, but unable for now because rescue places have put adoptions on hold.”*

(female, 45–54)

One participant reported she had considered fostering during the lockdown period. However, the restrictions in place would make it difficult to obtain a number of essentials required to care for her companion animal.

*“I have read that animal rescues have been inundated with offers to foster and adopt, but adoption is not possible. I have thought about fostering but the lockdown makes it difficult to get the things I would need for this, e.g., dog bed or crate, toys, dog bowls, collar, leash, etc.”*

(female, 55–64)

##### Reduced Provision of Animal-Assisted Interventions during COVID-19

It was frequently reported that animal-assisted interventions were no longer being delivered due to the COVID-19 lockdown. Participants reported that this had a detrimental effect not only on those receiving the intervention, but also what they perceived as a detrimental effect on the animals involved in the sessions. A few participants expressed that their animals were missing the interaction with those they usually work with.

*“Two of my dogs are therapy dogs who cannot visit due to COVID-19. They are really missing their work and interaction with other people.”*

(female, 55–64)

*“My pet therapy dog is missing cheering people up.”*

(female, 45–54)

*“My cat is a registered therapy cat. We are missing our visits; we are looking forward to returning to visit his fans. He is missing all his worship and fuss.”*

(female, 45–54)

### 3.3. Do Reported Changes Relating to Animal Welfare and Behaviour Differ by Animal Species? (RQ3)

The PCA reduced the 17 statements relating to changes in animals’ welfare and behaviour to two components: PC1 (positive changes in animal welfare and behaviour) and PC2 (negative changes in animal welfare and behaviour; see Table 4). Excluded statements that did not load > 0.4 were: ‘my animal has gained weight’, ‘my animal is following me around less’, ‘my animal has lost weight’, ‘my animal is more wary or hostile’, and ‘other welfare or behaviour change’.

Dogs and cats were the most commonly owned species (69.9%; n = 3719 and 44%; n = 2430, respectively), and those most commonly perceived to be owner’s closest companion animal. For those who identified owning “other species” (3.3%; n = 177), 22.0% (n = 39) considered these companion animals to be their closest animal. Since birds, fish, reptiles, and amphibians were less commonly considered as closest animals, they were collapsed into one category “non-mammals”, in order to have their PC scores compared to other species. Likewise, farm animals and “other species” were considered as “others”.

**Figure 1:**
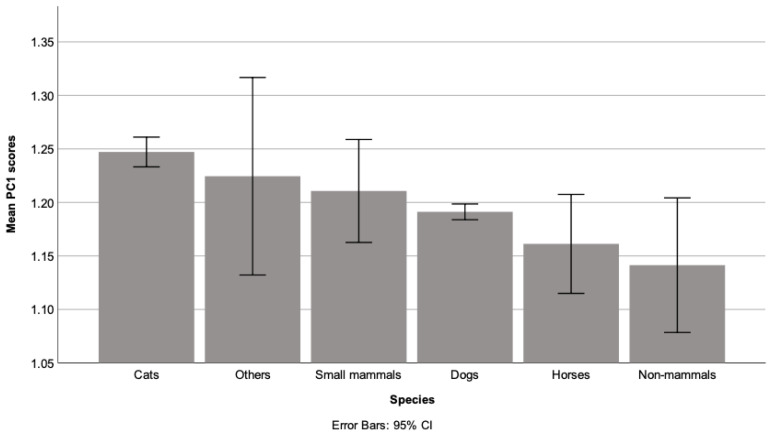
Bar graph of mean PC1 scores (positive animal welfare and behaviour changes) grouped by species.

A non-parametric one-way MANOVA was performed and showed that both PC scores significantly differed between the six animal species. For PC1 (positive reported changes; Figure 1), post-hoc tests showed the scores were significantly higher for cats compared with the following species: dogs (*p* = 0.001), non-mammals (*p* = 0.015) and horses (*p* = 0.021).

**Figure 2:**
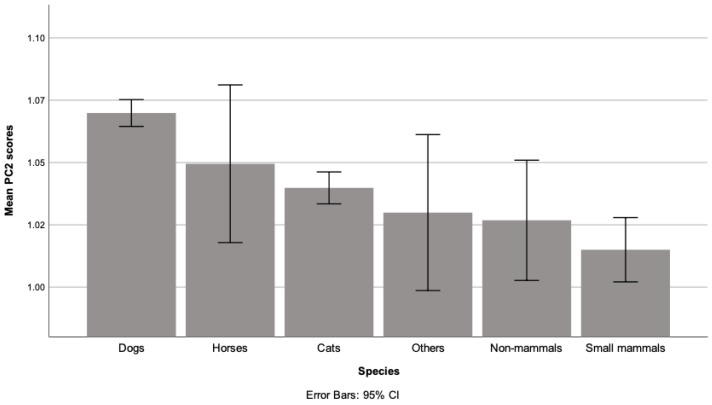
Bar graph of mean PC2 scores (negative animal welfare and behaviour changes) grouped by species.

For PC2 (negative reported changes; Figure 2), post-hoc tests showed these scores were significantly higher for dogs compared with the following species: cats (*p* = 0.001) and small mammals (*p* = 0.001).

### 3.4. What Is the Association between Companion Animal Owners’ Mental Health during the First UK COVID-19 Lockdown and the Reported Changes Relating to Their Animal’s Welfare and Behaviours? (RQ4)

Adjusting for relevant covariates, poorer mental health scores since lockdown were significantly associated with more reported positive changes in companion animal welfare and behaviour. However, mental health scores pre-lockdown were not significantly associated with reported positive changes in companion animal welfare and behaviour (see Table 5). Younger age, animal role (no role vs. working dog), stronger human-animal bond (as measured by the CCA), and not perceiving a companion animal as a family member were associated with reported positive changes in companion animals’ welfare and behaviour.

Poorer mental health scores before and since the COVID-19 lockdown phase had significant but opposite associations with the reported negative changes in companion animal welfare and behaviour. Adjusting for relevant covariates, poorer mental health scores pre-lockdown were significantly associated with fewer reported negative changes in companion animal welfare and behaviour. However, owners reported more negative changes in welfare and behaviour if they had poorer mental health scores since lockdown. Animal role (no role vs. assistance dog) was also associated with reported negative changes in companion animals’ welfare and behaviour.

## 4. Discussion

This mixed-method study explored the perceptions of companion animal welfare and behaviour change, together with their association with the companion animal owner’s mental health. To date, studies that have considered the impact of the COVID-19 pandemic on companion animals have tended to focus on detrimental effects [8], and on specific species (primarily dogs) [9]. Little attention has been paid to the overall impact on the spectrum of companion animals with whom we share our lives.

### 4.1. Reported Changes in Animal Welfare and Behaviour across a Range of Species

Our findings not only reinforce some of the concerns previously described for dogs, such as changes in exercise patterns [9] and exacerbation of current behaviour problems [8], but extend these concerns to other species. There are some remarkable consistencies in some of the reported concerns in companion animal welfare and behaviour. For example, for owners of dogs, cats and horses, approximately 10% (±2%) reported their companion animal to be more unsettled; 5.5% (±1.5%) reported their companion animal was more anxious; and 3.5% (±1.5%) reported their companion animal was more withdrawn.

However, there are also marked differences between species. Approximately a third of companion animal owners reported their cats and dogs increased following behaviour, and this was approximately twice the value of other species, who would generally be considered less accustomed to free movement within the home. Additionally, the behaviour of a greater proportion of horses, ponies and farm animals appeared to be unaffected. Some of these differences are unsurprising, given their lack of cohabitation with their owners, but perhaps what is more remarkable is that nearly 60% of the larger companion animals considered in this study were perceived to have changed their behaviour. Horses might have been particularly affected by changes in their routine, such as restrictions to riding during the first lockdown phase, aligning with previous studies investigating the impact of the pandemic on horses [25].

Overall, approximately a third of cats and dogs were reported to be unaffected by the first lockdown compared to around 40% of other species, and many animals appeared to have improved welfare as a result. Between 10–15% of all owners reported that their animal appeared to be more energetic and playful, and 20–30% indicated their animal seemed more relaxed; with at least three times as many owners reporting improvements rather than deteriorations in their animal’s physical condition. Our findings indicate that cats generally showed more positive signs of improved welfare (Figure 1). By contrast, dogs appeared to fare significantly worse than cats and small mammals (Figure 2), although it should be noted that the median value for the negative welfare component indicated no change. Thus, while there are undoubted concerns for specific animals (as indicated by the free-text comments), the impact of the pandemic on companion animals should not be portrayed as universally detrimental to them in general.

An unexpected finding was that a higher proportion of cat owners (35.9%) reported their companion animal was more affectionate during the lockdown phase compared to owners of other species. It has previously been suggested that what might appear to be increased attachment in cats may actually be reinforced resource-seeking behaviour [26]. There is evidence of specific owner reinforcement of cat vocalisation [27], and the current findings are consistent with cat social behaviour being sensitive to instrumental reinforcement. It is clear that many owners have a greater need for their animals’ company during the pandemic [3,6], with various forms of physical contact being particularly important [7]. Thus, it seems reasonable to suggest that the perceived increase in cat’s affectionate behaviour and dependence on the owner may be the result of changes in owner behaviour during lockdown, associated with an increased need for company and close physical contact.

### 4.2. Association between Reported Animal Welfare and Behaviour Changes and Owner’s Mental Health

Owner mental health status had a clear, albeit small, effect on perceptions of companion animal welfare and behaviour. Interestingly, our models indicate the reported positive changes were affected by mental health score since lockdown, but not pre-lockdown, and this differentiation deserves further consideration. The mental health score since lockdown indicates the status of the owner at the point of survey completion, and so might reflect the current needs of the owner at that timepoint, and how the individual was coping. Our results indicate that either the welfare and behaviour of companion animals improved as a result of a deterioration in owner health, or that the owner’s perception of their animal’s welfare and behaviour improved in these circumstances. Various forms of animal-assisted intervention have been reported to increase human empathy [28,29,30] and perceived social support may not only reduce stress, but also increase empathy [31]. Thus, it might be that those in greatest need for social support, as evidenced by poorer mental health scores since lockdown, are more empathic towards their animals’ needs. This may be particularly the case if the companion animal is the owner’s primary source of support, and it is perhaps unsurprising that the strength of the bond had a positive contribution in the final model.

Mental health scores since lockdown were also a predictor of reported negative changes in companion animal welfare and behaviour, albeit a negative one. While the negative influence of mental health scores since lockdown on the perceived negative welfare and behaviour changes might be explained in relation to the hypothesis outlined above, mental health scores pre-lockdown had the opposite effect. The pre-lockdown mental health score reflects the general state of the owner and is perhaps more closely related to the normal living standards and background that may have shaped the relationship between the owner and their companion animal. This also highlights the point described above, as poorer mental health may increase attention paid to one’s companion animal, and empathic engagement may increase reporting of any changes, both positive and negative, in animal welfare and behaviour.

However, relationships between owner mental health and perceived companion animal welfare and behaviour have been recognised for some time [32,33]. Severe trauma and depression in owners may in fact predict the subsequent development of problem behaviours in dogs [34]. More recently, in cats, an association has been identified between owner personality and animal health/wellbeing which shows some parallels with that observed between a carer and their child [35]. Clearly some mental health problems may directly result in poor animal welfare [36,37], but our results suggest poorer owner mental health may also affect perceived companion animal welfare and behaviour through less direct routes. Our findings extend previous species-specific concerns to a wide range of species.

### 4.3. Limitations

Firstly, the study population was a convenience sample that is not representative of the UK population, as the participants were predominantly female companion animal owners, a bias that is commonly cited in the field of human-animal interaction research [38]. Secondly, it is likely that many respondents frequently worked outside of the home prior to the pandemic, and knowledge of their companion animal’s behaviour at home before the lockdown phase may not have been extensive. Therefore, it may be challenging for the owner to interpret any changes in their animal’s welfare and behaviour in the pandemic context. Additionally, these changes were self-reported; they are not objective and may reflect the companion owner’s state of mind rather than actual changes in welfare and behaviour. Lastly, while a PCA was conducted to reduce the number of behaviour and welfare responses into a workable construct, this was interpreted subjectively. For example, perceived increased appetite could be a sign of stress and underlying health problems [39].

## 5. Conclusions

In conclusion, our study provided insight into the reported changes in companion animal welfare and behaviour, and the links between these changes and companion animal owners’ mental health. It extended previous insights into perceived welfare and behaviour changes of a range of companion animals, rather than focusing on one specific species. Our study also highlighted that mental health status had a clear, albeit small, effect on companion animal welfare and behaviour.

## Figures and Tables

**Table 1:** Participant characteristics for companion animal owners (n = 5323).

Characteristics		% (N)
**Gender**	Female	79.3 (4222)
	Male	20.0 (1062)
	Other	0.5 (30)
	Prefer not to say	0.2 (9)
**Age (years)**	18–24	6.4 (341)
	25–34	16.4 (871)
	35–44	16.5 (880)
	45–54	24.8 (1319)
	55–64	23.0 (1225)
	65–70	7.2 (384)
	Over 70	5.7 (303)
**Ethnicity**	White	97.3 (5179)
	Mixed/multiple ethnic	0.9 (50)
	Asian/Asian British	0.4 (22)
	Black/African/Caribbean/Black British	0.1 (6)
	Chinese	0.2 (8)
	Arab	0.0 (1)
	Other ethnic	0.2 (11)
	Prefer not to say	0.9 (46)
**Companion animal species**	Dogs	69.9 (3719)
	Cats	44.0 (2340)
	Small mammals	9.8 (519)
	Fish	9.1 (485)
	Horses or ponies	6.3 (334)
	Birds	5.3 (282)
	Reptiles	3.9 (208)
	Farm animals	1.2 (65)
	Amphibians	0.7 (37)
	Other	3.3 (177)
**Animals with a special role**	Emotional support animals	4.7 (251)
	Therapy animals	2.3 (123)
	Assistance dogs (e.g., guide dogs)	1.1 (57)
	Working dogs	0.9 (50)

**Table 2:** Reported changes in companion animal’s welfare and behaviour during the first COVID-19 lockdown phase, grouped by species.

	All Species (n = 5323)	Dogs (n = 3508)	Cats (n = 1469)	Horses, Ponies and Farm Animals (n = 97)	Other (n = 249)
	Agree % (N)	Agree % (N)	Agree % (N)	Agree % (N)	Agree % (N)
My animal is following me around more	33.1% (1764)	33.9% (1190)	35.3% (519)	17.5% (17)	15.3% (38)
There have been no changes in my animal’s behaviour	32.7% (1740)	32.7% (1149)	32.3% (474)	41.2% (40)	30.9% (77)
My animal is more affectionate	27.5% (1465)	24.8% (871)	35.9% (527)	19.6% (19)	19.3% (48)
My animal seems more relaxed	25.5% (1357)	25.3% (888)	27.2% (400)	27.8% (27)	16.9% (42)
My animal seems more energetic or playful than before	14.6% (777)	15.1% (530)	14.2% (208)	10.3% (10)	11.6% (29)
My animal is more sociable than before	13.8% (732)	8.4% (295)	25.2% (370)	7.2% (7)	24.1% (60)
My animal seems more unsettled	10.9% (584)	12.9% (454)	7.8% (115)	8.2% (8)	2.8% (7)
My animal’s appetite seems to have increased	9.1% (486)	8.2% (288)	12.2% (179)	1.0% (1)	7.2% (18)
My animal’s physical condition (e.g., coats/feathers) seems to have improved	7.8% (414)	7.7% (270)	7.6% (112)	17.5% (17)	6.0% (15)
My animal seems more anxious/easily scared	5.9% (314)	7.0% (244)	4.1% (60)	4.1% (4)	2.4% (6)
My animal has gained weight	5.2% (277)	5.6% (195)	3.9% (58)	15.5% (15)	3.6% (9)
My animal is quieter/more withdrawn than before	3.9% (212)	5.0% (177)	2.0% (29)	4.1% (4)	0.8% (2)
My animal is following me around less	2.8% (150)	3.1% (108)	2.6% (39)	2.1% (2)	0.4% (1)
My animal has lost weight	2.3% (125)	2.6% (92)	1.8% (26)	3.0% (3)	1.6% (4)
My animal has lost their appetite	2.3% (120)	2.7% (93)	1.8% (26)	0% (0)	0.4% (1)
My animal’s physical condition (e.g., coat/feathers) seems to have worsened	1.9% (103)	2.2% (77)	1.4% (20)	4.1% (4)	0.8% (2)
My animal is more wary or hostile towards me or family members than before	0.6% (34)	0.4% (15)	1.1% (16)	2.1% (2)	0.4% (1)

**Table 3:** Themes and associated sub-themes.

**Theme one: Positive impact on companion animals during COVID-19** Improvement in animal’s behaviour and temperament Improvement in animal’s physical condition
**Theme two: Negative impact on companion animals during COVID-19** Concerns over changes in animal’s temperament Concerns over changes in animal’s physical condition
**Theme three: Broader impact of COVID-19 on animal welfare** Negative impact of dog walking restrictions Adoption and fostering considerations and concerns Reduced provision of animal-assisted interventions during COVID-19

**Table 4:** Component pattern and component values.

	Components	
**Welfare and behaviour items**	**PC1**	PC2
More affectionate	**0.72**	0.04
More social	**0.66**	−0.1
More relaxed	**0.62**	−0.24
Follows owner more	**0.59**	0.3
More energetic	**0.56**	−0.01
Improved physical condition	**0.5**	−0.06
Increased appetite	**0.41**	0.12
More unsettled	−0.02	**0.74**
More anxious	0	**0.69**
Quieter/more withdrawn	−0.07	**0.61**
Worse physical condition	0.02	**0.45**
Decreased appetite	0.03	**0.41**
**Eigen values**	2.4	1.93
**Proportion variance**	0.2	0.16
**Total variance explained**	0.2	0.36
**Coefficient alpha**	0.67	0.56
**Kaiser-Meyer-Olkin index = 0.73**		

The values represent the loading of each item. Loadings of 0.4 or above are in bold.

**Table 5:** Generalised linear model for each PC scale and predictors (mental health score pre-lockdown and since lockdown) adjusting for relevant covariates.

PC1 (Positive Changes)		B	SE	*t*-Value	*p*-Value
(Intercept)		0.414	0.033	12.522	0.001 *
Mental health score pre-lockdown		−0.005	0.001	−0.316	0.752
Mental health score since lockdown		−0.010	0.001	−6.478	0.001 *
Age		−0.020	0.002	−10.576	0.001 *
Gender	Male vs. female	−0.003	0.007	−0.399	0.690
	Male vs. non-binary	−0.013	0.032	−0.422	0.673
Animal role	No role vs. emotional support	0.014	0.013	1.134	0.257
	No role vs. assistance dog	0.016	0.026	0.615	0.539
	No role vs. therapy animal	−0.022	0.018	−1.238	0.216
	No role vs. working dog	−0.017	0.029	−2.972	0.003 *
Human-animal bond		0.003	0.001	5.346	0.001 *
Perceiving companion animal as a family member		−0.019	0.006	−2.972	0.003 *
**PC2 (Negative Changes)**		**B**	**SE**	***t*** **-value**	***p*** **-value**
(Intercept)		0.102	0.024	4.192	0.001 *
Mental health score pre-lockdown		0.002	0.001	2.380	0.017 *
Mental health score since lockdown		−0.006	0.001	−5.110	0.001 *
Age		−0.002	0.001	−0.205	0.838
Gender	Male vs. female	0.009	0.005	1.841	0.066
	Male vs. non-binary	0.048	0.023	2.077	0.038
Animal role	No role vs. emotional support	0.015	0.009	1.566	0.117
	No role vs. assistance dog	0.052	0.019	2.710	0.007 *
	No role vs. therapy animal	0.022	0.013	1.707	0.088
	No role vs. working dog	0.038	0.021	1.780	0.075
Human-animal bond		−0.002	0.005	−0.423	0.672
Perceiving companion animal as a family member		0.005	0.005	1.009	0.313

Categorical variable: each row refers to one category compared to the reference category (left of the vs.). * indicates significance (*p* < 0.05)

## Data Availability

The data presented in this study are openly available on the OSF repository via the following URL: https://osf.io/4vefu/ (accessed on 2 February 2021).

## Supplementary Materials

The following are available online at https://www.mdpi.com/article/10.3390/ijerph18116171/s1, Table S1: Measure for perceived changes in companion animal’s welfare and behaviour.
