# Supplementary material for: The Perceived Impact of The First UK COVID-19 Lockdown on Companion Animal Welfare and Behaviour: A Mixed-Method Study of Associations with Owner Mental Health
Source: Int J Environ Res Public Health. 2021 Jun 7;18(11):6171. doi: 10.3390/ijerph18116171 (PMC8201214; doi:10.3390/ijerph18116171)
Supplement: Supplementary file 1 [file ijerph-18-06171-s001.zip › ijerph-1246688-supplementary.pdf]

Supplementary Table S1. Measure of perceived changes in companion animal's welfare and behaviour

**Have you noticed any of the following changes in your animal's behaviour since the social distancing measures started? Mark all that apply.**	My animal seems more relaxed
	My animal seems more unsettled
	My animal is more affectionate
	My animal is more sociable than before
	My animal is quieter/more withdrawn than before
	My animal is more wary or hostile towards me or family members than before
	My animal seems more anxious/easily scared
	My animal is following me around more
	My animal is following me around less
	My animal seems more energetic or playful than before
	My animal's appetite seems to have increased
	My animal has lost its appetite
	My animal has lost weight
	My animal has gained weight
	My animal's physical condition (e.g. coat/feathers) seems to have improved
	My animal's physical condition (e.g. coat/feathers) seems to have worsened
	Other (please specify): ___________________
	There have been no changes in my animal's behaviour
